# Elucidation of immunological response and its regulatory network by P-TUFT-ALT-2: a promising fusion protein vaccine for human lymphatic filariasis

**DOI:** 10.1098/rsos.172039

**Published:** 2018-05-16

**Authors:** Rajkumar Paul, Sandeep Jaiswal, Natarajan Mahalakshmi, Perumal Kaliraj

**Affiliations:** Centre for Biotechnology, Anna University, Sardar Patel Road, Guindy, Chennai 600025, Tamil Nadu, India

**Keywords:** abundant larval transcript-2, Tuftsin, human lymphatic filariasis, recombinant vaccine, real-time polymerase chain reaction

## Abstract

Human lymphatic filariasis, a mosquito-borne neglected tropical parasitic disease, needs an early development of prophylactic agents such as a vaccine for its successful elimination. Our earlier study suggested the enhanced immunological response by fusion protein (P-TUFT-ALT-2) of Tuftsin and ALT-2 in a mice model. We cultured human peripheral blood mononuclear cells (PBMCs) and treated cells with *Escherichia coli*-expressed ALT-2 (E-ALT-2) and P-TUFT-ALT-2. Real-time polymerase chain reaction was performed to identify the mRNA copy number of various cytokine and transcription factor genes. The recombinant vaccine candidate was also validated for humans by immunoreactivity with human sera samples of natural infection. In this study, P-TUFT-ALT-2 stimulated 12% higher PBMC proliferation in endemic normal (EN) individuals than E-ALT-2 alone. There was enhanced production of IFN *γ*, IL-2, IL-5 and IL-12, indicating a balanced Th1/Th2 response. However, higher expression of IL-5 and lower IL-4 validate the humoral response through an IL-5-dependent manner. Also, high level of IL-17 indicates a strong Th/Treg regulation over T-cell activation. The upregulated T-bet might have enhanced IFN-γ production, whereas GATA-3 was supposed to enhance IL-5 expression. The fusion protein also exhibited 15–16% higher reactivity with EN clinical sera, exposing the upregulation of IgG1 and IgM in natural infection. The higher reactivity of P-TUFT-ALT-2 with sera of natural infection (EN) was validated indirectly by B-cell activation through various cytokines and regulatory genes produced from different T cells. Thus, these findings endorse P-TUFT-ALT-2 as a potential vaccine candidate for human lymphatic filariasis.

## 1. Introduction

Lymphatic filariasis is a mosquito-borne parasitic disease caused mainly by *Wuchereria bancrofti* and *Brugia malayi.* The filarial parasites have already caused lymphatic filariasis in 947 million people of 54 countries, with over 36 million critically disfigured [1]. About 57% of the world's population faces the risk of infection with lymphatic filarial parasites in tropical regions of southeast Asia (nine countries), and about 37% of these people live in the African region (35 countries). India shares about 30% of the disease burden in the whole world [2]. The Ministry of Health and Family Welfare, Government of India joined with the Global Network for Neglected Tropical Diseases (Global Network), an initiative of the Sabin Vaccine Institute, to direct the development of a branded public service advertising campaign named ‘Hathipaon Mukt Bharat’ (Filaria-Free India) in close coordination with the National Vector Borne Disease Control Program [3]. The World Health Organization has already initiated a Global Program to Eliminate Lymphatic Filariasis (GPELF) by the year 2020 with mass drug administration using diethylcarbamazine or ivermectin combined with albendazole [4–6]. Anti-parasitic drugs are successful to control parasitic diseases. However, these have only microfilaricidal effect without any impact on the larval or adult worms even after prolonged application. Hence, control of the disease requires effective vaccine development approaches along with drugs [7,8]. According to various research reports, L3 infective stage-specific ALT-2 is the most abundant and it showed remarkable immune response in mice and immunoreactivity in human clinical samples [9–16]. People infected with *B. malayi* show a high frequency of IgG1 and IgG3 antibodies to ALT-2 [17].

Tuftsin, a natural tetrapeptide of threonine–lysine–proline–arginine is a well-recognized potential immuno-potentiator to enhance the immunogenic response of various antigens [18]. It enhances immunogenicity of an antigenic protein by exposing it to antigen-presenting cells, such as macrophages and dendritic cells producing elevated humoral and cellular immune responses [19,20]. P-TUFT-ALT-2 (fusion protein of Tuftsin and ALT-2) had already shown enhanced immunogenic response in an experimental mice model in our previous study (R.P. unpublished data). The vaccine works in the human body by regulating expression of various cytokines and regulatory genes. T-cell proliferation and differentiation are dependent on cytokines. Finding out the underlying mechanism of vaccine response will help in vaccine formulation and its proper therapeutic application with other drugs, adjuvants or immuno-modulator.

The cytotoxic T lymphocyte antigen 4 (CTLA-4) expressed in Tregs and upregulated T-cells regulates T-cell activation, Th1/Th2 differentiation and tolerance [21]. The forkhead box P3 (*Foxp3*) or Scurfin secreted from Th1 cells enhances the production of Treg cells. The transcription factor GATA-binding protein 3 (GATA3) is an important regulator of the differentiation of Th0 cells towards the Th2-cell subtype, promoting the secretion of IL-4, IL-5 and IL-13 from Th2 cells, while it suppresses the differentiation towards Th1 cells [22]. The induced T-cell co-stimulatory gene (ICOS) on Th2 cells produces humoral immune responses. The Th1 cell-specific T-box transcription factor TBX21 (T-bet) is expressed in dendritic cells, natural killer cells, natural killer T cells, CD4^+^ and CD8^+^ T effector cells, B cells, *γδ* T cells and T_Reg_ cells [23]. Transforming growth factor beta (TGF-β) secreted by white blood cells and macrophages induces apoptosis or programmed cell death in lymphocytes [24]. The RAR-related orphan receptor gamma (ROR*γ*) is expressed in immature CD4^+^/CD8^+^ T cells and helps in the differentiation of pro-inflammatory Th17 cells and expression of IL-17 [25].

In the present study, we have examined the P-TUFT-ALT-2-mediated expression pattern of various cytokines and regulatory genes involved in immunoregulation. We also verified the immunoreactivity of the P-TUFT-ALT-2 fusion protein with human clinical sera samples of natural infection to establish a correlation between cell-mediated and humoral immune response.

## 2. Material and methods

### 2.1. Expression and purification of P-TUFT-ALT-2

*Pichia pastoris/tuft-alt-2* and *P. pastoris/alt-2* having 11 copies of gene construct was cultured in 100 ml of buffered glycerol-complex medium and incubated for 48 h until optical density (OD) was 6 at 600 nm. The culture was spun and resuspended in 200 ml of buffered minimal methanol yeast medium and incubated for 72 h at 24°C and 200 r.p.m. with 1% methanol induction after every 24 h. The culture broth was spun at 10 000 r.p.m. for 10 min and the supernatant was collected. The supernatant was mixed with 70% ammonium sulfate overnight at 4°C. The precipitated protein was dialysed with a 10 kDa dialysis membrane (HiMedia, India) by changing the dialysis buffer after every 4 h for 12 h. The dialysed sample was dissolved in loading buffer (20 mM Tris and 5 mM EDTA, pH 8.0) containing 200 mM NaCl and passed through ion exchange chromatography with the matrix Q-Sepharose™ fast flow (GE Healthcare, Danderyd, Sweden). The semi-purified sample was further purified in size exclusion chromatography with the Superdex™ 75 matrix (GE Healthcare) using 20 mM Tris (pH 8.0) buffer containing 150 mM NaCl.

*Escherichia coli*-expressed ALT-2 (E-ALT-2) was expressed in Luria Bertani broth from *E. coli* BL21 (DE3) pLysS under T7 promoter in pRSETB vector induced with 1 mM IPTG for 3 h at 37°C and 150 r.p.m. in a shaker. The E-ALT-2 was purified in immobilized metal affinity chromatography using 250 mM imidazole.

### 2.2. Ethical statement for human blood sample

Human blood samples were collected after obtaining the informed consent from all persons with permission (reference no. 26433/VCII/SI/2012) of the Department of Public Health and Preventive Medicine, Government of Tamil Nadu. All methods were carried out in accordance with relevant guidelines and regulations issued by the same department. All experimental protocols were approved by the institutional review board at the Centre for Biotechnology, Anna University, India. The thick smear slide method was used to differentiate various groups of filarial patients based on recommendation by the Tamil Nadu Government (table 1). Blood samples from asymptomatic microfilaraemics (MF) (*n* = 10) were collected from an endemic region of Tamil Nadu, India after the patients were identified as per the traditional thick smear microscopic data provided by the Department of Public Health and Preventive Medicine and Zonal Entomology Team, Vellore. Endemic normal (EN) (*n* = 10) samples were collected from individuals of the endemic villages having no mf present in the thick smear microscopic test, but having circulating antigens. The samples of chronic pathology (CP) (*n* = 10) with visible clinical symptoms of lymphedema were collected from Vellore and Tiruvannamalai districts. The control non-endemic normal (NEN) sera were provided by Professor Murray Selkrirk, Imperial College London from a non-endemic region in the United Kingdom.

**Table 1.** Demographic and haematological parameters.

parameters	group	endemic normal	microfilaria	chronic pathology
sex	male	4	5	3
	female	6	5	7
age	<20 years	1	0	0
	20–40 years	4	2	0
	40–60 years	4	6	7
	>60 years	1	2	3

### 2.3. Human peripheral blood mononuclear cell proliferation assay

Peripheral blood mononuclear cells (PBMCs) were isolated from 10 ml of heparinized venous blood collected from each EN individual (*n* = 10) by gradient centrifugation over lymphocyte medium (Lymphoprep; Nycomed Pharma AS, Oslo, Norway) at 2000 r.p.m. for 15 min. All the procedures were performed in a laminar hood. The cells were washed with RPMI-1640 medium containing antibiotics twice followed by centrifugation at 1500 r.p.m. for 10 min. One millilitre of the same medium having 10% heat-inactivated fetal calf serum (FCS) was added to the cells and the viability was determined by the trypan blue dye (1 : 1) exclusion method under a microscope.

The cells were then cultured in round-bottomed microtitre plates at a concentration of 0.2 × 10^6^ cells well^−1^ in RPMI 1640 with 1% antimycolytic, 25 mM HEPES, 2 mM glutamine and 10% heat-inactivated FCS. The cells were stimulated with E-ALT-2, P-ALT-2 and P-TUFT-ALT-2 (each of 1 µg ml^−1^), soluble crude extract of L3 larvae (10 µg ml^−1^) and positive control ConA (10 µg ml^−1^). Cells with medium alone were used as unstimulated controls. The cultures were incubated at 5% CO_2_ and 37°C for 72 h. Alamar blue dye was added to the culture (10 µl well^−1^) after 64 h of culture and incubated for another 8 h. The reading was taken at OD_570_. The proliferative responses were expressed as the stimulation index (SI) calculated as cells stimulated with antigen divided by unstimulated cells [26]. All cultures were taken in triplicate and the results expressed as mean SI ± s.e.m.

**Table:** 

real-time PCR genes		
gene name	forward primer (5′–3′)	reverse primer (5′–3′)
*β-actin*	AAGAGCTACGAGCTGCCTGAC	GTAGTTTCGTGGATGCCACAG
*IFN-γ*	AGCTCTGCATCGTTTTGGGTT	GTTCCATTATCCGCTACATCTGAA
*IL-2*	CCAGGATGCTCACATTTAAGTTTTAC	TTTGAGTTCTTCTTCTAGACACTGA
*IL-4*	AGCCTCACAGAGCAGAAGAC	GCCCTGCAGAAGGTTTCCTT
*IL-5*	AGCTGCCTACGTGTATGCCA	GCAGTGCCAAGGTCTCTTTC
*IL-10*	TGTCATCGATTTCTTCCCTGTGAA	TCTTGGAGCTTATTAAAGGCATTCTT
*IL-12*	CAGAAGCTAACCATCTCCTGGTTTG	CCGGAGTAATTTGGTGCTCCACAC
*IL-17*	TGGGAAGACCTCATTGGTGT	GGATTTCGTGGGATTGTGAT
*CTLA-4*	ATCTGCAAGGTGGAGCTCAT	AATCTGGGTTCCGTTGCCTA
*TGF-β*	ACTACTACGCCAAGGAGGTCAC	CTGCTTGAACTTGTCATAGATTTCG
*ICOS*	GGATGCATACTTATTTGTTGGCTTA	TGTATTCACCGTTAGGGTCGT
*T-bet*	GTTCCCATTCCTGTCCTTC	CCTTGTTGTTGGTGAGCTT
*GATA-3*	GAACCGGCCCCTCATTAA	ATTTTTCGGTTTCTGGTCTGGAT
*Foxp3*	CAGCACATTCCCAGAGTTCCTC	GCGTGTGAACCAGTGGTAGATC
*RORγt*	CGCTCCAACATCTTCTCC	CTAACCAGCACCACTTCC

### 2.4. Isolation of mRNA and cDNA preparation

After collecting supernatants for the cytokine assay, Trisol (Sigma-Aldrich, St Louis, MO, USA) was added to the pelleted splenocyte cells (4 × 10^6^ cells ml^−1^) and stored at −80°C. Then, cells were lysed using the reagents of a commercial kit (QIAshredder; Qiagen, Spoorstrat, Venlo, The Netherlands), and total RNA was isolated according to the manufacturer's protocol in RNase-free water (RNAiso Plus, TAKARA, Japan). The cDNA was prepared by reverse transcription polymerase chain reaction (PCR) with 0.4 mM of each dNTP, 0.4 lg of random hexamers, 200 U Moloney murine leukaemia virus reverse transcriptase (New England BioLabs Inc, Ipswich, MA, USA) at 42°C for 60 min, 50 mM Tris–HCl (pH 8.3), 3 mM MgCl_2_ and 62.5 mM KCl.

### 2.5. Real-time polymerase chain reaction

The quantitative PCR analysis was performed in the StepOne Real-Time ABI 7500 sequence detection system (Applied Biosystems, CA, USA) using SYBR™ Master mix dye to determine the mRNA copy number of cytokine genes such as IL-2, IL-4, IL-5, IL-10, IL-12, IL-17 and IFN *γ*. We also performed real-time PCR to determine the mRNA copy number of regulatory genes like *TGF-β, CTLA-4, ICOS, T-bet*, *GATA-3*, *Foxp3* and *RORγt* with *β-actin* as the reference gene copy number. Each reaction mix (10 µl) contained 5 µl of 2× SYBR master mix, 0.4 µl of each of 250 nM forward and reverse primers and 1 µl of 1 : 10 diluted cDNA template. The initial denaturation was at 95°C for 10 min followed by 40 cycles of 95°C for 15 s, 60°C for 1 min, 72°C for 30 s and finally 72°C for 10 min. The cDNA from unstimulated PBMC cultures were used as control. *ARG4* target was used as endogenous controls for copy number and gene expression experiments. The data collection of the fluorescence signal was performed after the elongation step. Relative transcript or mRNA copy number was determined by 2^−ΔΔCT^, in which CT is the threshold cycle during the exponential phase of amplification along with the 25th, 50th and 75th percentile distribution of mRNA copy number [27]. The following primers were used in real-time PCR amplification.

### 2.6. Indirect ELISA for total sera IgG

The E-ALT-2, P-ALT-2 and P-TUFT-ALT-2 antigens (100 ng well^−1^) were diluted in coating buffer containing 0.1 M bicarbonate (pH 9.6). The antigens were coated in 96-well plates (Nunc Maxisorp, Nalge Nunc International, Denmark) followed by incubation overnight at 4°C. The plates were blocked with 5% skimmed milk powder at 37°C for 1 h after washing three times with PBS-T. Human clinical sera of MF, CP, EN and NEN were diluted in PBS-T (1 : 100). The diluted sera were added to the wells (100 µl well^−1^) and kept in incubation at 37°C for 1 h. The goat anti-human IgG alkaline phosphatase conjugate (Sigma-Aldrich) diluted at 1 : 1000 in PBS-T was added (100 µl well^−1^) after washing thrice with PBS-T. The plates were kept in incubation again at 37°C for 1 h. Plates were washed thoroughly thrice with PBS-T. The mixture of *p*-nitrophenyl phosphate disodium salt (pNPP) (Sigma-Aldrich) was used as substrate (1 mg ml^−1^) in substrate buffer (NaHCO_3_—0.84 g l^−1^; Na_2_CO_3_—1.25 g l^−1^; MgCl_2_—0.2 g l^−1^). The substrate solution was added to the wells and incubated for 20–30 min. The absorbance was measured at 405 nm in the ELISA reader (BioTek Instruments, Inc., USA).

### 2.7. Isotype ELISA

The plates were coated with the antigens, i.e. E-ALT-2, P-ALT-2 and P-TUFT-ALT-2 (100 ng well^−1^). The human clinical samples were used as the primary antibody as described above. The mouse anti-human IgG isotypes (IgGl [1 : 500], IgG2 [1 : 2000], IgG3 [1 : 5000] and IgG4 [l : 5000]) were added as secondary antibodies (Sigma-Aldrich) after washing and incubation at 37°C for 1 h. Again, after washing the plates with PBS-T followed by PBS, goat anti-mouse IgG–ALP conjugate (Sigma-Aldrich) was added at a 1 : 1000 dilution and incubated at 37°C for 1 h. The plates were then washed three times with PBS-T. About 100 µl of pNPP substrate was added (Sigma-Aldrich). The OD was read at 405 nm after 20–30 min.

## 3. Statistical analysis

All statistical analyses were done using GraphPad Prism software version 6.0. The non-parametric Kruskal–Wallis test was used for multiple comparisons. For immunoreactivity and T-cell proliferation studies, two-way ANOVA was used to determine the differences among groups. A probability *p*-value < 0.05 was considered statistically significant.

## 4. Results

### 4.1. Peripheral blood mononuclear cell proliferation assay

**Figure 1.**
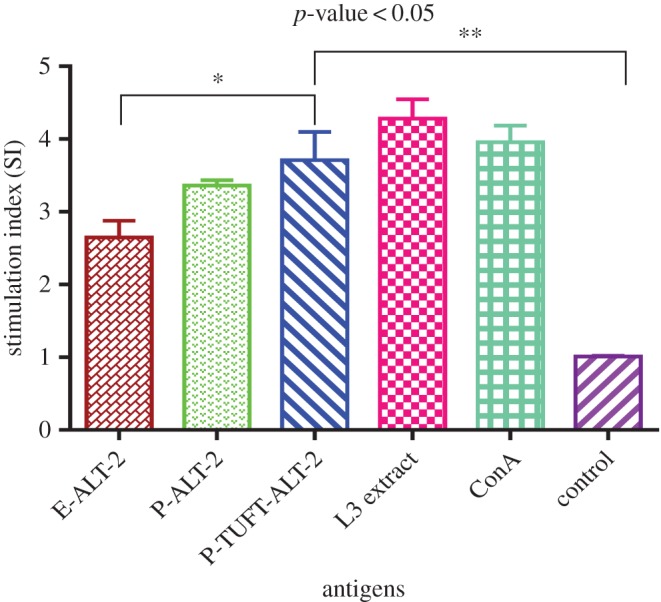
Proliferation of human EN PBMCs stimulated with antigens. Proliferation of human PBMCs was stimulated by E-ALT-2, P-ALT-2 and P-TUFT-ALT-2. Con A was used as a positive control and cell with medium alone was used as control. The data are represented as the mean stimulation index (SI) of 10 EN individual samples ± s.e.m. The asterisk on top of the bars indicates a significantly high SI value for P-TUFT-ALT-2 compared to E-ALT-2 (*p* < 0.05) and control cells (*p* < 0.05) and control (*p* < 0.001). Unpaired *t*-test with Welch's correction was performed.

The PBMC proliferation of 10 EN samples was carried out to analyse the T-cell response induced by P-ALT-2 and P-TUFT-ALT-2 in humans (figure 1). The P-TUFT-ALT-2 fusion protein showed significantly high (*p* < 0.02) proliferation (SI = 3.71 ± 0.23) compared to E-ALT-2 protein (SI = 2.65 ± 0.13) and P-ALT-2 (SI = 3.36 ± 0.04). The crude extract of L3 antigen was used as a positive control that showed high proliferation (mean SI = 4.28 ± 0.16) over ConA (mean SI = 3.96 ± 0.13) and control (mean SI = 1.01 ± 0.01).

### 4.2. Cytokine analysis

**Figure 2.**
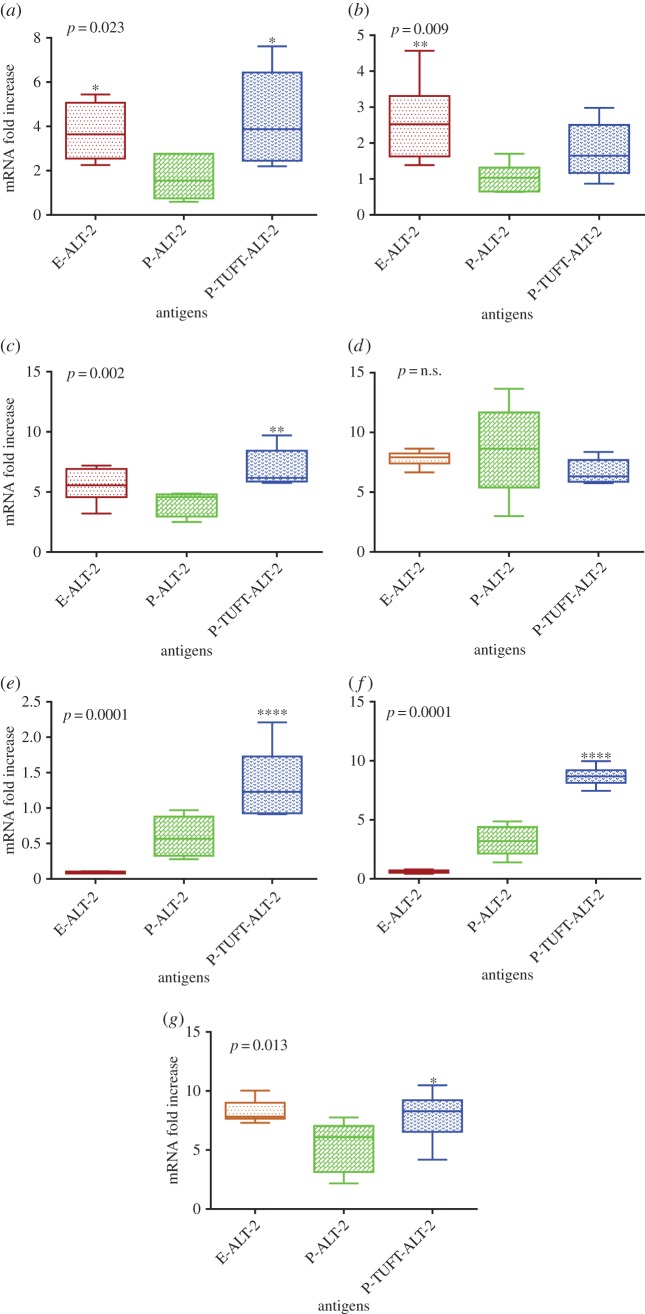
Expression of cytokines in PBMCs stimulated by filarial antigens. PBMC expression of (*a*) IL-2, (*b*) IL-4, (*c*) IL-5, (*d*) IL-10, (*e*) IL-12, (*f*) IL-17 and (*g*) IFN-γ mRNA is depicted as fold change following 48 h stimulation with E-ALT-2, P-ALT-2 and P-TUFT-ALT-2 vaccine antigens. Result expressed as box and whisker plots; horizontal lines represent the 25th, 50th and 75th percentiles; vertical lines represent the 10th and 90th percentiles of data. Values of *p* were calculated using the Kruskal–Wallis test.

The IL-2 and IFN-γ levels were high in the cultures stimulated with P-TUFT-ALT-2 (3.88- and 7.89-fold) and E-ALT-2 (3.64- and 8.22-fold). However, the mRNA fold numbers of IL-5, IL-12 and IL-17 were significantly higher in P-TUFT-ALT-2-induced PBMCs (6.96-, 1.35- and 8.60-fold, respectively) than in the E-ALT-2-induced culture (5.56-, 0.09- and 0.62-fold, respectively). The high level of IL-10 (seven- to ninefold) was observed in all cases (figure 2*a–g*).

### 4.3. Regulatory gene expression profile

**Figure 3.**
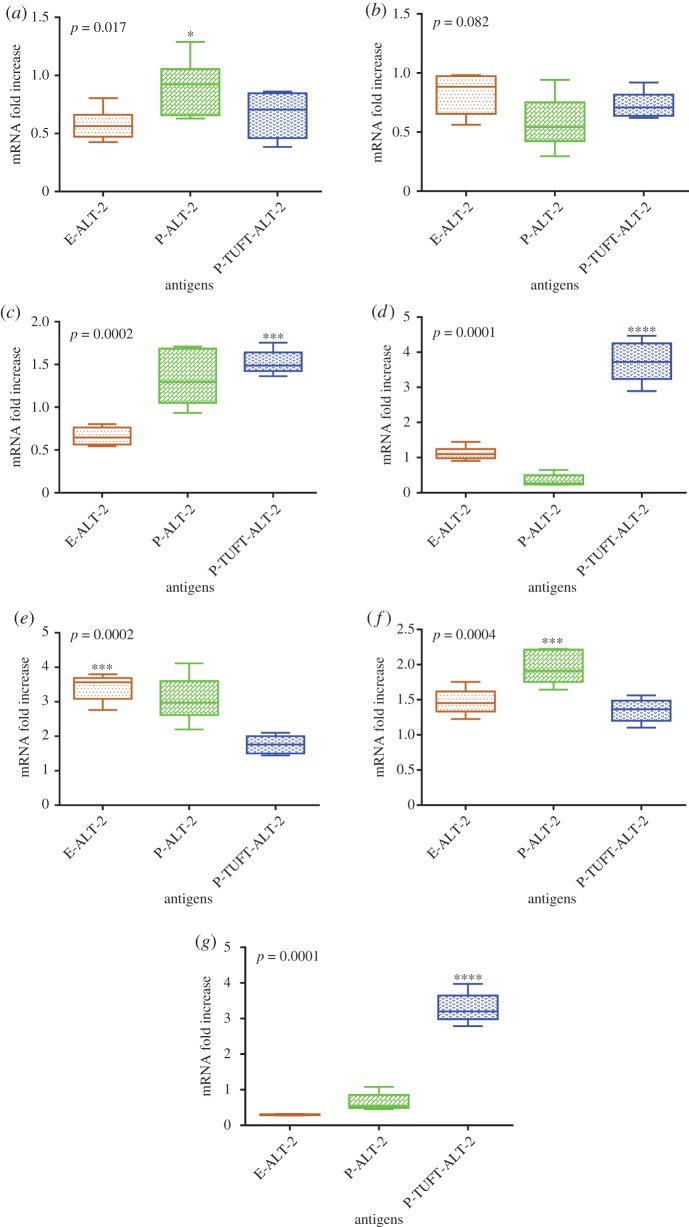
Expression of regulatory genes in filarial infection. PBMC expression of (*a*) CTLA-4, (*b*) FoxP-3, (*c*) GATA-3, (*d*) ICOS, (*e*) Tbet, (*f*) TGF-β and (*g*) ROR*γ*t mRNA following 48 h stimulation with E-LAT-2, P-ALT-2 and P-TUFT-ALT-2 vaccine antigens depicted as the fold change over medium control. Result expressed as box plots; horizontal lines represent the 25th, 50th and 75th percentiles; vertical lines represent the 10th and 90th percentiles of data. Values of *p* were calculated using the Kruskal–Wallis test.

The *GATA-3* gene was expressed in E-ALT-2 (0.66-fold), but it was upregulated in P-ALT-2 (1.29-fold) and P-TUFT-ALT-2 (1.56-fold). *T-bet* was seen as highly upregulated in both E-ALT-2 (3.42-fold) and P-ALT-2 (3.17-fold) compared to P-TUFT-ALT-2 (1.76-fold). *ICOS* and *RORγt* genes were observed to be highly upregulated in P-TUFT-ALT-2 (3.72-fold and 3.30-fold) compared to E-ALT-2 (1.12-fold and 0.29-fold) and P-ALT-2 (0.35-fold and 0.64-fold) (figure 3*a–g*).

### 4.4. Indirect ELISA for total IgG

**Figure 4.**
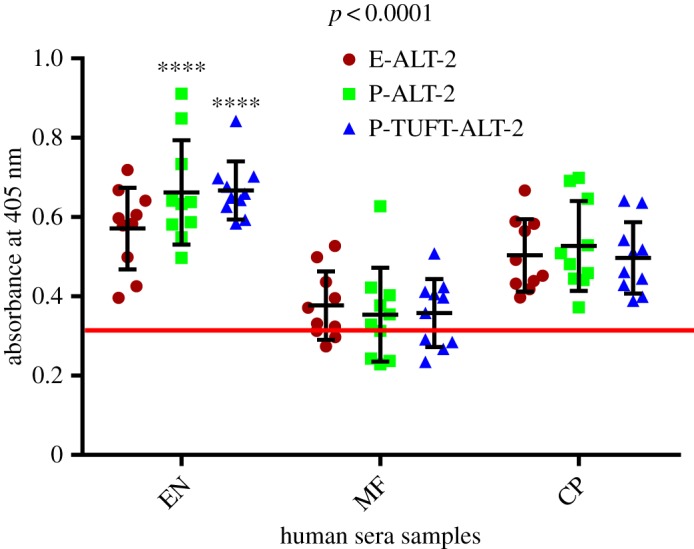
Total IgG antibody titre against E-ALT-2, P-ALT-2 and P-TUFT-ALT-2 in EN, MF and CP human sera samples. Indirect ELISA was carried out with EN, MF and CP sera (*n* = 10) from an endemic region of Tamil Nadu, India. Data are represented as an aligned scatter plot where each symbol represents absorbance of individual sera and the horizontal line in each group represents the mean OD. The mean absorbance ± 3 s.d. of the negative control was considered. Two-way ANOVA was performed to determine the differences among the groups (*p* < 0.0001). NEN sera was considered as the cut-off value, which is represented as the horizontal line parallel to the *X* axis.

**Figure 5.**
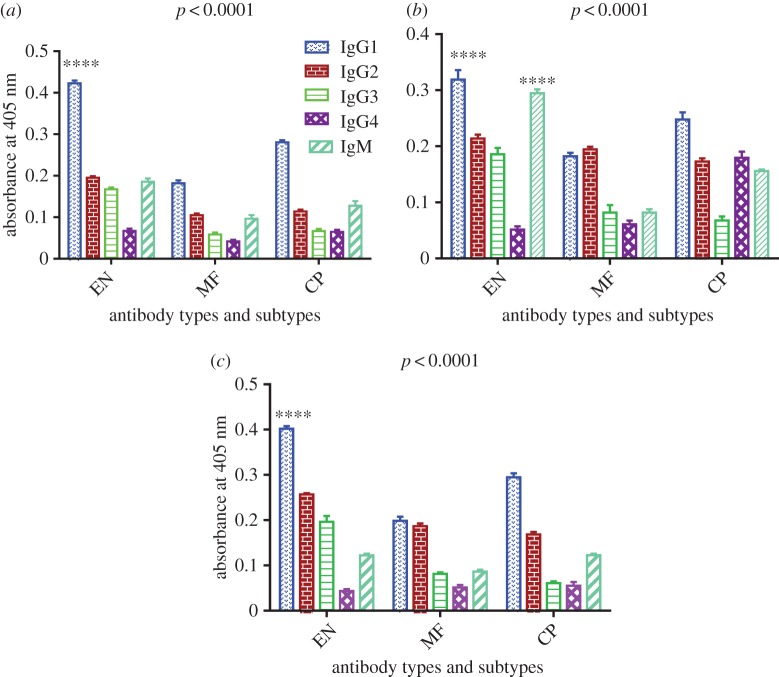
Subtypes of antibody titre in human sera. (*a*) E-ALT-2, (*b*) P-ALT-2, (*c*) P-TUFT-ALT-2: IgG1, IgG2, IgG3 and IgG4 isotypes along with IgM levels against recombinant proteins were determined by indirect ELISA in the individual sera of different clinical groups in endemic areas of Tamil Nadu, south India. Data represent the mean absorbance of ten samples ± s.e.m. *Two-way ANOVA was performed to determine the difference among the groups, *p* < 0.0001.

The levels of antigenicity exhibited by P-ALT-2 with human EN, MF and CP sera were 0.66 ± 0.04, 0.35 ± 0.04 and 0.53 ± 0.04, respectively (*p* < 0.0001) at mean OD_405._ This was better than that of E-ALT-2 (mean OD_405_ = 0.57 ± 0.03, 0.37 ± 0.03, 0.50 ± 0.03, respectively (*p* < 0.001)) (figure 4). The P-TUFT-ALT-2 also showed significantly higher reactivity with sera (mean OD = 0.67 ± 0.02, 0.36 ± 0.03 and 0.49 ± 0.03), which was similar to that of P-ALT-2, but higher than that of E-ALT-2 sera. Importantly, about 15–16% higher reactivity was observed in P-ALT-2 and P-TUFT-ALT-2 with EN sera (mean OD_405_ = 0.66 ± 0.04 and 0.67 ± 0.02) (figure 5).

### 4.5. Isotype ELISA

The P-TUFT-ALT-2 showed IgG1 (0.42 ± 0.01), IgG2 (0.20 ± 0.01), IgG3 (0.17 ± 0.01), IgG4 (0.07 ± 0.01) and IgM (0.19 ± 0.01), whereas P-ALT-2 showed IgG1 (0.32 ± 0.03), IgG2 (0.21 ± 0.01), IgG3 (0.19 ± 0.01), IgG4 (0.05 ± 0.01) and IgM (0.30 ± 0.01) in EN sera samples. E-ALT-2 also indicated IgG1 (0.40 ± 0.01), IgG2 (0.26 ± 0.01), IgG3 (0.20 ± 0.02), IgG4 (0.04 ± 0.01) and IgM (0.12 ± 0.01) in the same samples. The IgG1 isotype was predominant followed by IgG2 and IgM in EN, MF and CP sera in all cases (figure 5*a–c*).

## 5. Discussion

The balance in the humoral and cellular arms of the immune system is essential to combat infection and, thus, any vaccine antigen should be capable of eliciting both antibody and T-cell responses in humans, especially in filariasis, since there is evidence of T-cell suppression immediately after infection [28]. The T epitopes of antigen were identified by stimulation of human PBMCs from EN exposed to the filarial antigens. The antigens carrying dominant T epitopes specific for human MHC molecules would bind MHCs in the antigen-presenting cells with high affinity, and induce T-cell proliferation *in vitro*. PBMCs from EN individuals have been reported to proliferate at varying degrees against crude filarial antigens [29], which correlates with our observation. Both EN and CP samples showed high proliferative response with crude *B. malayi* adult antigens (BmA), whereas MF individuals showed poor or no proliferation consistent with previous observations [30]. P-TUFT-ALT-2 showed elevated cellular hyper-response and the lymphocyte proliferation of EN PBMCs to filarial antigens *in vitro,* releasing high levels of IFN-γ and IL-5. Anti-inflammatory cytokines IL-10 and TGF-β are also released. The highest IFN-γ secretion could enhance cellular responses in filarial infection. Tuftsin-based fusion proteins significantly improved the phagocytotic activity of macrophages along with suppression of human epidermoid carcinoma growth [20]. The protective immune response against many helminth parasites has been referred to as the type 2 response, which is characterized by increased levels of IL-4, IL-5, IL-9, IL-13 and IL-21, activation and expansion of CD4^+^ Th2 cells, plasma cells secreting IgE, eosinophils, mast cells and basophils [28,31]. The Th2 type of response was marked by IgG1 isotype and secretion of IL-5 cytokine. Both IL-4 and IL-5 cytokines were shown to be essential for protection along with antibodies. The higher IL-5 production by P-TUFT-ALT-2 was independent of IL-4. IL-5 has been demonstrated directly to be essential for protection induced by irradiated L3 larvae and for resolution of primary infection in a fully permissive murine filariasis [31]. According to various reports, IL-5 is involved in immunity to filarial nematodes [28,32]. IL-12 is best known for its capacity to polarize Th1 cells and to activate NK and NKT cells. IL-12 is thus primarily involved in the initiation of cellular immune responses against intracellular pathogens. The high level of IL-10 confirms the presence of immunomodulatory domains in P-TUFT-ALT-2. In our experiment, the elevated level of suppressive IL-10 might be involved in T-cell regulatory functions via Th17 cell activation producing high level of regulatory IL-17. IL-17 is also associated with the Th2-type response and can promote Th2-cell differentiation and nematode parasite expulsion. As parasite clearance is attributed mainly to type 2 responses in filariasis [28], this should enhance the protection against filarial infection. In a similar study, a series of recombinant proteins containing malaria B and T epitopes in various combinations cloned in hepatitis B virus core protein were assayed for immunogenicity in mice and showed protective immunity against malaria [33]. The high IL-2 was responsible for PBMC proliferation in this study.

The increased expression levels of circulating T cells expressing the inhibitory marker CTLA-4 was observed in filarial infection [20]. Diminished levels of another immunosuppressive mediator, TGF-β, was observed in nodules around parasites [34]. The inhibition of binding of CTLA-4 to its ligands increased the production of IL-2, IL-3 and IFN-γ by Th1 cells, and IL-3, IL-4, IL-5 and IL-10 by Th2 cells [35]. Therefore, low expression of CTLA-4 by P-TUFT-ALT-2 increased IL-2, IL-5, IL-10 and IFN-γ. T-cell suppression mainly depends on TGF-β, not IL-10 and CTLA-4 [36]. FoxP3 suppresses the function of NF-kB, leading to suppression of the expression of IL-2 and effector T-cell cytokines [37]. FoxP3 also functions as a transcription activator for CTLA-4, but the knocking down of FoxP3 does not affect CTLA-4 expression [38]. Interaction of FoxP3 with ROR-γt inhibits IL-17 production in Th17 cells. FoxP3 is highly expressed in activated Th17 cells and contributed to their phenotype by suppressing IFN-γ production [39]. There was a lower expression of *CTLA-4* and *FoxP-3* and higher expression of *TGF-β* in all cases. Gao *et al.* [24] reported the decreased expression in forkhead/winged helix transcription factor p-3 (Foxp-3) as well as CTLA-4, and secretion of TGF-β in PBMC culture stimulated with tuftsin. Foxp3 transcription factor represses transcription of key genes involved in regulatory T-cell function by occupying the promoters following stimulation of T-cell receptors [40]. TGF-β and IL-21 can convert the FOXP3+ T regulatory cells into Th17 cells. The lower expression of FoxP-3 by P-TUFT-ALT-2 might not suppress IL-2 and IL-17, and was not able to activate CTLA-4. The activated ICOS−/− CD4^+^ T cells have reduced IL-4 secretion, but similar IFN-γ secretion and IL-5 as observed in our experiment. T-bet acts on genes with roles in transcriptional regulation such as that of *BCL6*, *ATF4*, *CREB1* and *IFI16,* and expresses *PTGER4*, *IL4*, *FOXO3A* and *CD28* [41]*.* TGF-β transforms Foxp3-positive regulatory T cells (iTregs) into IL-17-producing Th17 cells in the presence of IL-6 [42]. TGF-β has also been shown to downregulate inflammatory cytokine production in monocytes and macrophages, by inhibition of NF-κB [43]. Here, elevated TGF-β enhanced Th2 cells to express ROR*γ*t. In this experiment, RORyt and T-bet were upregulated, indicating Th17 cell proliferation and IL-17-mediated immune response.

As the epitopes recognized in natural infection are important, the reactivity of the peptides was tested by ELISA with 10 human sera samples from each of the clinical groups, i.e. EN, MF, CP and NEN, in order to identify the B epitopes recognized in humans, which may vary from that of mice. Identification of reactivity of ALT-2, recognized in natural infection of humans, has practical implications in the understanding and prevention of disease. Many filarial proteins including secreted larval acidic protein 1 (SLAP1) from *Onchocerca volvulus*, an ALT-2 homologue, have been characterized [44]. A chimeric protein construct comprising thioredoxin, transglutaminase and ALT-2 showed enhanced immunological response [45]. Any potential vaccine must be able to elicit antibody expression that recognizes the native antigen. The *Pichia*-expressed P-ALT-2 and P-TUFT-ALT-2 exhibited higher affinity with EN sera. This indicates that the P-ALT-2- and P-TUFT-ALT-2-induced immune system would clear filarial pathogens by the ADCC mechanism with greater potentiality. The IgG1 isotype was predominant followed by IgG2 and IgM. Both IgG1 and IgG3 can bind to Fc*γ*RI on the surface of various effector cells, activating them to kill *B. malayi* larvae [46–48]. In a similar study, the BmHsp12.6*α*c subunit of the BmHsp12.6 protein showed a higher level of IgG1 and IgG3 antibodies in putatively immune EN sera, which are involved in the ADCC-mediated protection, suggesting it to be a potential vaccine candidate [49]. IgM is the only isotype that reacts strongly with the surface of *Brugia* L3 [50]. IgG4 makes 95% of the blood immunoglobulin in MF patients, and IgE levels are raised in patients with CP and EN [51]. EN has IgE levels as high as CP, which suggests that IgE may not be the factor responsible for pathology. High levels of filarial-specific IgG1, IgG2, IgE and IgM and low level of IgG4 were observed for P-TUFT-ALT-2. This further confirms the possibility of modulating early immune response in the host as IgG4 secretion induced by Tr-1 cells in an IL-10-dependent mechanism is known to downregulate the immune response in MF patients [52–54]. Several other reports in both humans and cats prove that IgE is required for adult filarial nematode killing. Most of the L3 surface reactive antibodies were IgM and IgG2 in human sera. The higher immune response in PBMCs by P-TUFT-ALT-2 over P-ALT-2 may be due to its proper presentation and processing with Tuftsin fusion. However, they did not vary much in human clinical sera samples for their similar epitopes. P-TUFT-ALT-2 also showed higher immune response over E-ALT-2, which might be due to its proper folding and configuration in the eukaryotic expression system.

## 6. Conclusion

From the above experiment we can conclude that the presence of T-cell receptors in P-TUFT-ALT-2 showed high proliferation of T cells producing various cytokines. P-TUFT-ALT-2 elicited 12% higher PBMC proliferation over the E-ALT-2 antigen alone by exposing T-cell epitopes. The IL-2 and IFN-γ levels were high in the cultures stimulated with P-TUFT-ALT-2 and E-ALT-2. However, the mRNA fold numbers of IL-5, IL-12 and IL-17 were significantly higher in P-TUFT-ALT-2-induced PBMCs than in the E-ALT-2-induced culture. The high level of IL-10 was observed in all cases. The fusion protein enhanced the production of cytokines like IFN *γ*, IL-2, IL-5, IL-12 and IL-17, indicating a balanced Th1/Th2 response. Various regulatory genes like T-bet, GATA-3, ICOS and RORyt were upregulated, whereas CTLA-4 and FoxP3 were downregulated, indicating Th17 cell-mediated immune response regulation. The P-TUFT-ALT-2 also showed significantly higher reactivity with naturally infected human sera samples, which was similar to P-ALT-2, but higher than that of E-ALT-2 compared to NEN control sera. Importantly, about 15–16% higher reactivity was observed in P-ALT-2 and P-TUFT-ALT-2 with EN sera. The IgG1 isotype was predominant followed by IgG2 and IgM. The higher production of IgG1, IgG2, IgM and IgE seemed to be mediated through an IL-5-dependent manner. Thus, P-TUFT-ALT-2 may be considered as a potential vaccine candidate for human lymphatic filariasis. The finding on the regulation of cytokines and regulatory factors by the P-TUFT-ALT-2 fusion protein may lead to the proper formulation of this vaccine with other adjuvant or drug or modulator for successful treatment of human lymphatic filariasis.

## Supplementary Material

Supplementary Materials

## Data accessibility

This article does not contain any additional data.
