# Supplementary material for: Elucidation of immunological response and its regulatory network by P-TUFT-ALT-2: a promising fusion protein vaccine for human lymphatic filariasis
Source: R Soc Open Sci. 2018 May 16;5(5):172039. doi: 10.1098/rsos.172039 (PMC5990782; doi:10.1098/rsos.172039)
Supplement: Supplementary Materials [file rsos172039supp1.doc]

**Supplementary Materials**

**5’ATGACTAAGCCACGTGGTAGTACTAAGCCACGTAGTATGAATAAACTTTTAATAGCATTCGGTTTGGTAATTCTTTTTGTGACACTTCCGTGTGCATCAGAATCAGACGAAGAGTTCGATGACTCCGCAGCCGATGACACCGACGACAGCGAGGACGGAGGTGGTAGTGAAGGAGGTGATGAATATGTAACCAAAGGAGAATTTGTTGAAACTGATGGCAAAAAGAAAGAGTGCTCTTCGCACGAAGCTTGCTACGATCAACGTGAACCACAAGCGTGGTGCAGACTGAGCGAGAATCAGGCATGGACTGACAGAGGCTGCTTCTGCGAAGATAAGTTGCATTCGTGCGTCATCGAAAGAACGAACAATGGTAAATTGGAGTATTCGTACTGTGCACCTGAAGCAGGTTGGCAATGCGCATAG-3’**

1. **a)**

**Met Thr Lys Pro Arg Gly Ser Thr Lys Pro Arg Ser Met Asn Lys Leu Leu Ile Ala Phe Gly Leu Val Ile Leu Phe Val Thr Leu Pro Cys Ala Ser Glu Ser Asp Glu Glu Phe Asp Asp Ser Ala Ala Asp Asp Thr Asp Asp Ser Glu Asp Gly Gly Gly Ser Glu Gly Gly Asp Glu Tyr Val Thr Lys Gly Glu Phe Val Glu Thr Asp Gly Lys Lys Lys Glu Cys Ser Ser His Glu Ala Cys Tyr Asp Gln Arg Glu Pro Gln Ala Trp Cys Arg Leu Ser Glu Asn Gln Ala Trp Thr Asp Arg Gly Cys Phe Cys Glu Asp Lys Leu His Ser Cys Val Ile Glu Arg Thr Asn Asn Gly Lys Leu Glu Tyr Ser Tyr Cys Ala Pro Glu Ala Gly Trp Gln Cys Ala Stop**

1. **b)**

**Figure 1** a). Nucleotide sequence of *alt-2* and tuftsin fusion construct b). amino acid sequence of TUFT-ALT-2 fusion protein. Skynlue shadow represents Tuftsin part in both cases.

**
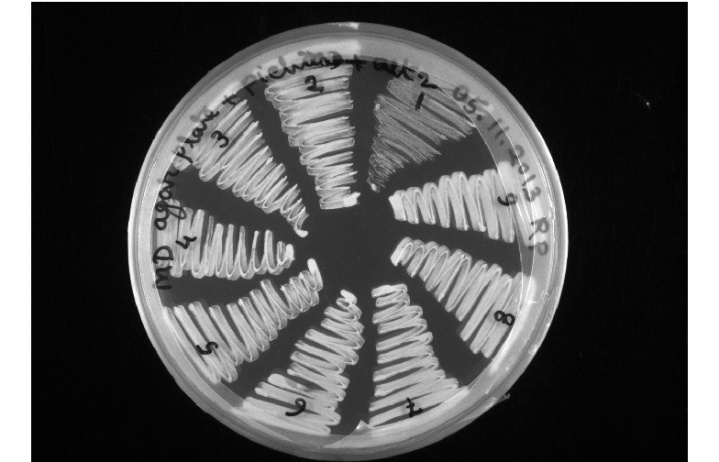

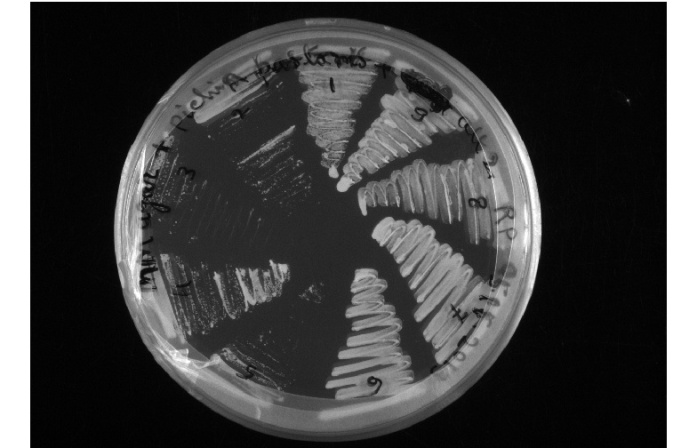
**

**1 b) i. 1b) ii.**

**Figure 2. (a) *Pichia pastoris/tuft-alt-2*** colonies grown on MD plate (b) ***P. pastoris/tuft-alt-2*** colonies grown on MM plate.


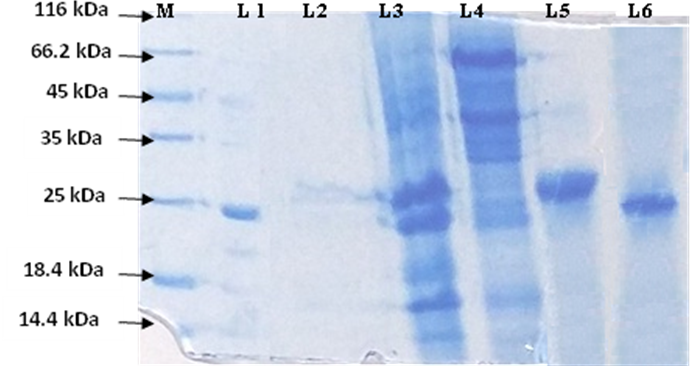


**Figure 3.** 12% SDS-PAGE of 10% TCA precipitated Supernatants of *P. pastoris*/*Bm alt-2* culture in BMMY: M : Protein Marker, L1 : Culture broth of Induced *P. pastoris/pPIC9K*, L2: Culture broth of uninduced *P. pastoris*/*tuft-alt-2,* L3 : Culture broth of Induced *P. pastoris*/*tuft-alt-2,* L4 : Cell lysate of Induced *P. pastoris*/*tuft-alt-2*, L5: Purified *Pichia* TUFT-ALT-2 L6: Purified *E. coli* ALT-2.
